# Editorial: Polydopamine-Based Nanostructures: Synthesis and Biomedical Applications

**DOI:** 10.3389/fchem.2020.00206

**Published:** 2020-03-20

**Authors:** Li Fu, Aimin Yu

**Affiliations:** ^1^College of Materials and Environmental Engineering, Hangzhou Dianzi University, Hangzhou, China; ^2^Department of Chemistry and Biotechnology, Faculty of Science, Engineering and Technology, Swinburne University of Technology, Hawthorn, VIC, Australia

**Keywords:** nanostructures, polydopamine, biomedical application, photosensitizer, host response diagnostics, drug delivery

Polydopamine (PDA) is a brown-black insoluble biopolymer with a melanin structure. Inspired by the powerful adhesion of invertebrate mussels, PDA came to public attention as a new coating material in 2007. One of the advantages of PDA coating is its mild and simple process which is based on the *in-situ* polymerization of its monomer dopamine (DA). It has been demonstrated that PDA can not only form uniform coatings on various organic and inorganic substrates including superhydrophobic surfaces, but also allow for further functionalization and secondary reactions. Due to its excellent biocompatibility and low cytotoxicity, PDA and its composite nanostructures have found many applications in various biomedical fields. This Research Topic collects a diverse range of fundamentals and applications of PDA, including nanomaterial synthesis, enzyme encapsulation, electrochemical sensing and antifungal agents.

The Research Topic contains two review articles. Xiong et al. summarized the application of PDA in photosensitizer. This is an important emerging area particularly for photodynamic therapy field. PDA-based materials are leveraged to overcome many most crucial shortcomings of current photosensitizer such as low light stability, rapid blood clearance and poor water solubility. The second review article wrote by Talon et al. focused on the use of PDA to improve host responses to polytetrafluoroethylene-based implants, specifically for the treatment of the Congenital Diaphragmatic Hernia. The PDA coating on the polytetrafluoroethylene surface showed incredible performance for cell attachment. Based on the *in vitro* evaluation, *in vivo* experiments in rats were recommended by authors to further confirm the results.

The three original research articles are showcases of three specific applications of DPA. The first work by Tran et al. reports the preparation of PDA hollow capsules by coating silica particles with PDA followed by dissolving silica template simply in water. Biomacromolecules such as catalase could be encapsulated into PDA hollow capsules with remained bio-functionality. This work provides an eco-friendly approach for hollow capsule fabrication which could be utilized as a drug delivery system. In the second work, Alves et al. investigated the antifungal performance of the polydimethylsiloxane after the surface modification of liposomal amphotericin embedded PDA. This work shows the liposomal amphotericin embedded PDA coating has an ability to prevent the attachment of *Candida albicans* and kill the adherent cells, without having toxic effect toward mammalian cells. The third work conducted by Fu et al. investigated the use of the PDA-functionalized graphene for the extraction of plant essential compounds for the electrochemical medical plant identification. The plant tissue wrapped by PDA-functionalized graphene has a very good film-forming property. Additionally, the PDA-functionalized graphene could adsorb and concentrate bioactive components from plant tissue and subsequently enhance the electrochemical signals.

The contributions in this Research Topic of Polydopamine-Based Nanostructures: Synthesis and Biomedical Applications highlight that manipulating PDA for surface modification enables to reach toward utilization. The applications covered in this Research Topic, supported by the state-of-the-art analysis and characterization, provide a dissection of the status of the field of the PDA-based biomedical applications. The *in vivo* study of the use of the PDA is worthy of exploring in the near future.

## Author Contributions

All authors listed have made a substantial, direct and intellectual contribution to the work, and approved it for publication.

### Conflict of Interest

The authors declare that the research was conducted in the absence of any commercial or financial relationships that could be construed as a potential conflict of interest.

